# Thoracic wall reconstruction with Collamend® in trauma: report of a case and review of the literature

**DOI:** 10.1186/1749-7922-7-39

**Published:** 2012-12-23

**Authors:** Federico Coccolini, Marco Lotti, Paolo Bertoli, Roberto Manfredi, Dario Piazzalunga, Stefano Magnone, Luca Campanati, Luca Ansaloni

**Affiliations:** 1 General and Emergency Surgery dept. Ospedali Riuniti, Largo Barozzi, 1-26128, Bergamo, Italy

**Keywords:** Biological prosthesis, Cross-linked, Thoracic, Reconstruction, Infected fields, Trauma, Collamend

## Abstract

**Introduction:**

Despite progress in reconstructive techniques, rebuilding portions of the thorax remains challenging, in particular when large resections, contamination or infection are involved. No other cases of thoracic reconstruction in trauma patients with biological prosthesis have been described since now.

**Methods:**

We report a case of thoracic reconstruction in highly infected field in a trauma patient. We also performed a literature review about the topic.

**Conclusion:**

Collamend® demonstrated its usefulness in thoracic wall reconstruction even in trauma patients and infected fields.

## Article

### Introduction

Thoracic wall (TW) reconstruction involves different surgical specialties as oncologic, plastic or trauma surgeon. Despite progress in reconstructive techniques, rebuilding portions of the thorax remains challenging, in particular when large resections, contamination or infection are involved. The successful reconstruction must preserve thoracic wall stability and respiratory function, eliminate dead spaces, avoid or reduce the risk of infection and protect the underlying viscera [1,2]. Indications for full thickness resection of thoracic wall are primary thoracic tumors, extensive extra-thoracic neoplastic diseases, congenital aplasia or traumatic events [3-5].

Beyond anatomical repair with soft tissue flaps and plastic surgery techniques many different prosthetic materials have been used for TW reconstruction. Polypropylene, polyester, expanded polytetrafluoroethylene (PTFE) and polyethylmethacrylate sandwiched between layers of polypropylene have been used [6]. In the last 15 years innovative materials have been introduced. Biological meshes comprised of several different materials: partially remodeling prosthesis are made of porcine dermal collagen (PDC), human dermal collagen (HDC), and bovine pericardium collagen (BPC). Completely remodeling prostheses are made of swine intestinal sub-mucosa (SIS), HDC and BDC. The partially remodeling prostheses are optimal in TW or abdominal reconstruction because of they resist better to mechanical stress. They are physically modified with cross-linkages between the collagen fibers which strengthen them [7,8].

In trauma surgery it often happens to stabilize thoracic wall injuries. Different techniques have been reported with different devices. The main challenge in trauma surgery is the potential contamination or the infection of the surgical field. One of the main characteristic of biological materials is the possibility to be used safely in contaminated or infected fields.

Biological prostheses have already demonstrated their usefulness and versatility in many fields [9-15]. However as the main part of literature is composed by case series and case reports, they still require more evidence-based data [16]. Recently a decisional model about the use of these mesh have been proposed by the Italian Biological Prosthesis Work Group [17]. In the last years the Italian Chapter of the European Hernia Society started the Italian Register of Biological Prosthesis (IRBP). Also the European Hernia Society, in the European Register of Biological Prosthesis (ERBP) is recruiting cases of biological meshes implanted all around Europe [16].

## Materials and methods

We report a case of TW reconstruction with Bard CollaMend® (Davol, Cranston, RI) in a patient victim of trauma.

A retrospective review was conducted of all reported cases of use of biological prosthesis in TW reconstruction in trauma published up to September, 2012 on PUBMED (1966–2012), using the key words, “thorax, reconstruction, biological, trauma”.

## Results

### Literature review

No other reports exist about the TW reconstruction in trauma with biological prosthesis.

### Case report

**Figure 1:**
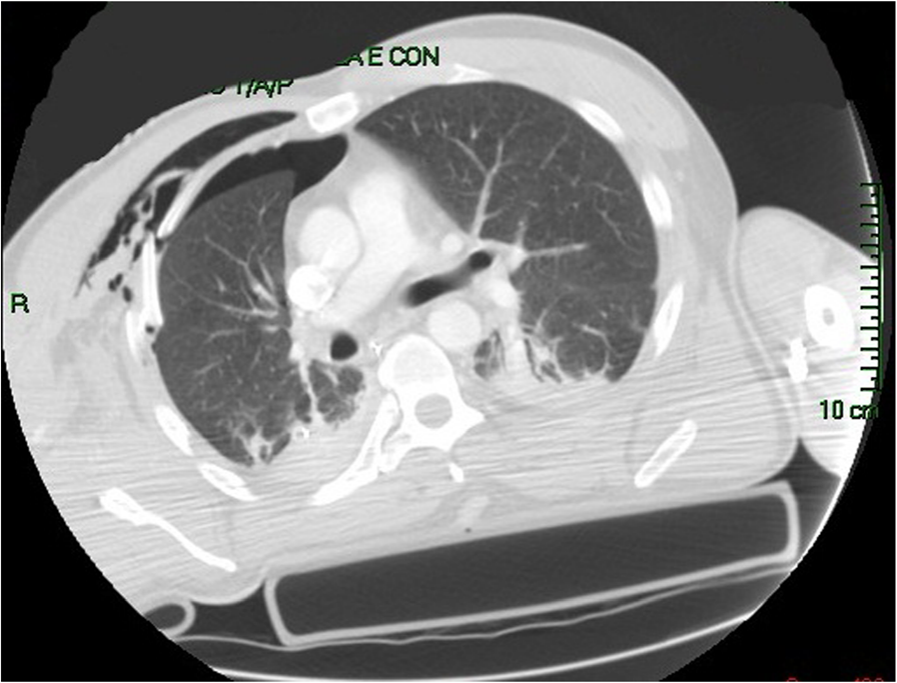
Pre-operative CT-scan.

**Figure 2:**
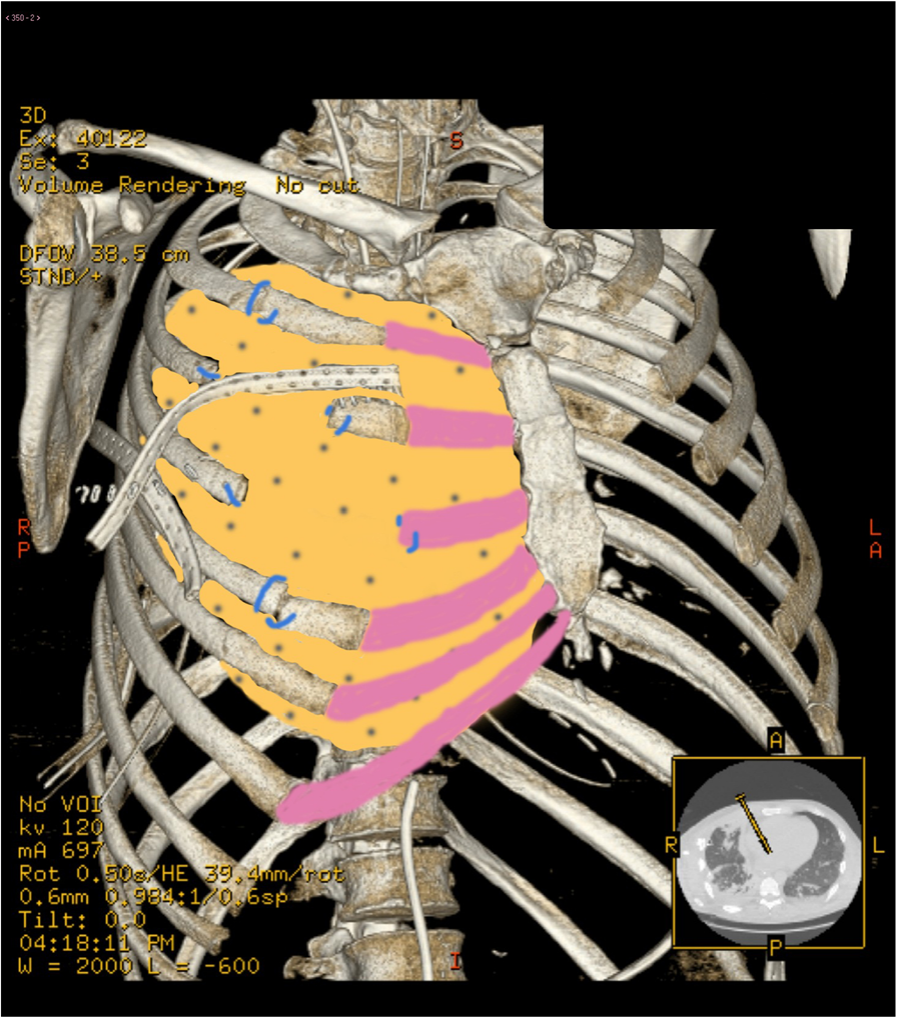
Reconstruction scheme with biological prosthesis.

**Figure 3:**
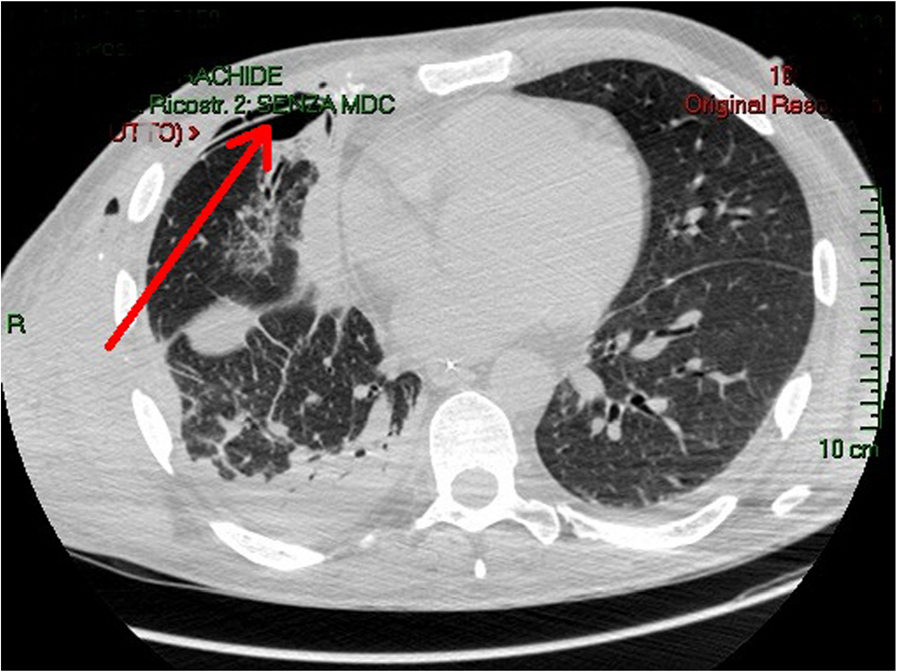
CT-scan 9 days after the reconstruction; the red arrow indicates the prosthesis.

**Figure 4:**
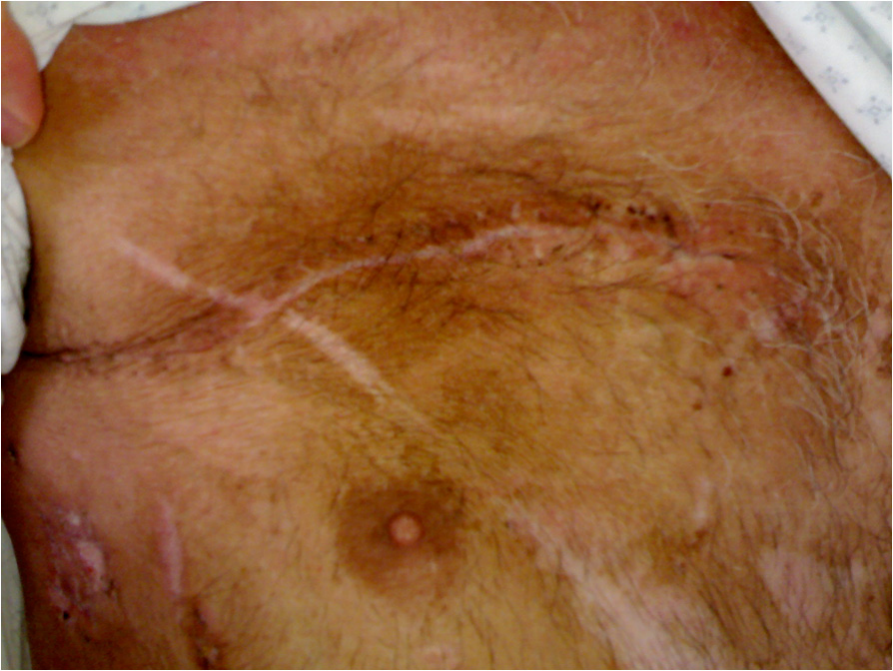
The complete healed thoracic wound.

A 47 years old male transported to the Emergency dept. of our hospital after a car crash. At the arrive in ER the patient was shocked with a bi-lateral pneumothorax, multiple rib fracture (II-III-IV-V-VI) with flail-chest on the right side (Figure 1), haemo-peritoneum and an exposed fracture of the right femur. Bilateral thoracic drains were immediately positioned and the patient was then transferred to the theatre for an explorative laparotomy and liver packing. Two days after the packing have been removed and the flail-chest (III and IV ribs) was fixed with titanium devices. The femur fracture was temporarily treated with external fixator. Ten days after the intervention the postoperative course was complicated by a biliary fistula treated with ERCP and biliary endo-prosthesis positioning. During the ICU recovery the patients developed ARDS and chest wound infection. Blood samples and chest wound cultures demonstrated infection by *Aspergillus Fumigatus* and *Pseudomonas Aeruginosa* MRSA respectively. The antibiotic treatment have been immediately addressed. 21 days after the intervention the patient have been re-operated for hemorrhagic shock from erosion of the right internal mammary artery by the rib margin. Surgical haemostasis was necessary. Free segment of the III and IV ribs were removed. Due to the infection titanium devices were removed too and the defect (7×8 cm) was repaired using a biologic mesh (CollaMend®, 18×23 cm) fixed to the thoracic wall with PDS-0 interrupted suture (Figure 2). 9 days after the second intervention a thoracic-abdominal CT-scan was performed (Figure 3). It documented no thoracic pathologic findings, satisfactory post-surgical results and a left hepatic artery post-traumatic pseudo-aneurism treated with angio-embolization. Femur fracture was then definitively treated with endomidollar pin positioning. Chest wound infection was treated with medication and healed completely in four weeks (Figure 4). 18 months after the discharge the patient is well and with documented no respiratory impairment.

## Discussion

The majority of patients were treated for TW reconstruction after surgical debulking procedure for thoracic malignancies or for extended abdominal tumors which interested the chest wall. TW reconstruction is a real challenge for thoracic surgeons as well. The reconstructive options are reduced under circumstances of potential of demonstrated wound infection. Biologic materials are specially indicated in potentially contaminated or contaminated surgical fields [18]. Their resistance to the proteases activity either bacterial either human is demonstrated. Moreover they have the unique characteristic to promote the early revascularization of the regenerate tissue. This allows to antibiotics to early reach the infected zone and by reducing the bacterial possibilities to create biologic niches as in synthetic prosthesis it favors the infection healing. A mild inflammatory response to these materials encourages active tissue deposition and natural cytokine production with a consequent healing process and tissue repair. As organized tissue deposition occurs, bio-scaffold is gradually remodeled by host, yielding a repaired tissue structure that is entirely host derived [14,19,20].

The challenge in TW reconstruction is the complex mechanisms involved in respiration. It implies muscular and elastic forces whom combined work results in the respiratory equilibrium. It briefly consists in a mild intra-thoracic negative pressure. A prosthetic material have to maintain this equilibrium constant to allow the patient to breath. It also has to avoid at the same time the air passage through the prosthesis preventing the subsequent pneumo-thorax. The alteration of the respiratory equilibrium results in either obstructive or restrictive impairment. Thoracic reconstructive materials must have either enough rigidity to allow the thorax to move symmetrically either elasticity to be able to adapt to the thorax movement. When a big portion of TW have to be removed and consequently many ribs lack, the reconstruction process risks to create an additional respiratory death space. Some reconstructive methods insert metal devices to guarantee the necessary rigidity. However if infection is suspected or demonstrated the insertion of a foreign body becomes a risky procedure.

In infected fields two are the possibilities: anatomic reconstruction with flap transposition or the use of biologics. The use of synthetic materials have been widely described with very good results, but in our opinion is very risky in potentially contaminated or infected fields. Reported side effects of synthetic materials include secondary wound infection in up to 6% of cases, seroma formation, insufficient tensile strength with respiratory failure, long-term onset of restrictive lung disease, graft dehiscence, chronic pain, hemorrhage and wall deformities in pediatric patients [3,21-23]. As counterpart, the experience in TW reconstruction with biologics is limited. Their use is progressively increasing and giving good results [24]. No other cases have been reported in literature of thoracic reconstruction in trauma patients. However in selected cases such a kind of materials could offers a very trustworthy alternative. The present case demonstrated the possibility to treat infections also by multi-resistant bacteria with the contemporary implantation of a biologic mesh. The described case was very challenging for the necessity to repair TW and the impossibility to implant foreign body. The *Pseudomonas Aeruginosa* MRSA infected wound, in fact reduced the therapeutic options. The patients needed a procedure as shorter and as less invasive as possible. He could hardly tolerate a long TW reconstructive procedure as in elective patients.

If biologics demonstrated to have usefulness properties, as counterpart the main obstacle to their use is the cost. It is absolutely higher than synthetic mesh, and in patients without infected or, at least potentially contaminated field the use of biologics have not a clearly stated rationale.

## Conclusions

Collamend® demonstrated its usefulness in thoracic wall reconstruction even in trauma patients and infected fields. Biological prosthesis confirmed to be a good alternative to synthetic materials either in reconstructive thoracic surgery. However dedicated studies from high experienced centers are needed.

## Competing interests

The Bard CollaMend® (Davol, Cranston, RI) mesh was purchased through the funds of the National Health System (Servizio Sanitario Nazionale). The authors have no conflict of interest and have the full control of the study and production of the present report.

## Authors’ contribution

FC, ML, LA participated in study design, literature search, data collection, manuscript writing, patient management and data analysis, RM, DP, SM, LC e PB participated in data interpretation, preparation of the figures and patient management. All authors read and approved the final manuscript.
